# Intact Fibroblast Growth Factor 23 Regulates Chronic Kidney Disease–Induced Myocardial Fibrosis by Activating the Sonic Hedgehog Signaling Pathway

**DOI:** 10.1161/JAHA.122.026365

**Published:** 2022-09-14

**Authors:** Lanlan Li, Hua Gan

**Affiliations:** ^1^ Department of Nephrology The First Affiliated Hospital of Chongqing Medical University Chongqing China

**Keywords:** cardiac fibroblasts, chronic kidney disease, intact fibroblast growth factor 23, myocardial fibrosis, Sonic Hedgehog signaling, Animal Models of Human Disease, Remodeling

## Abstract

**Background:**

Clinically, myocardial fibrosis is one of the most common complications caused by chronic kidney disease (CKD). However, the potential mechanisms of CKD‐induced myocardial fibrosis have not been clarified.

**Methods and Results:**

In our in vivo study, a rat model of CKD with 5/6 nephrectomy was established. The CKD model was treated with the glioma 1 (Gli‐1) inhibitor GANT‐61, and myocardial fibrosis and serum intact fibroblast growth factor 23 levels were assessed 16 weeks after nephrectomy. Finally, we found that Gli‐1 and Smoothened in the Sonic Hedgehog (Shh) signaling pathway were activated and that collagen‐1 and collagen‐3, which constitute the fibrotic index, were expressed in CKD myocardial tissue. After administering the Gli‐1 inhibitor GANT‐61, the degree of myocardial fibrosis was reduced, and Gli‐1 expression was also inhibited. We also measured blood pressure, cardiac biomarkers, and other indicators in rats and performed hematoxylin‐eosin staining of myocardial tissue. Furthermore, in vitro studies showed that intact fibroblast growth factor 23 promoted cardiac fibroblast proliferation and transdifferentiation into myofibroblasts by activating the Shh signaling pathway, thereby promoting cardiac fibrosis, as manifested by increased expression of the Shh, Patch 1, and Gli‐1 mRNAs and Shh, Smoothened, and Gli‐1 proteins in the Shh signaling pathway. The protein and mRNA levels of other fibrosis indicators, such as α‐smooth muscle actin, which are also markers of transdifferentiation, collagen‐1, and collagen‐3, were increased.

**Conclusions:**

On the basis of these results, intact fibroblast growth factor 23 promotes CKD‐induced myocardial fibrosis by activating the Shh signaling pathway.

**Nonstandard Abbreviations and Acronyms**
5/6NX: 5/6 nephrectomy
α‐SMA: α‐smooth muscle actin
ECM: extracellular matrix
FGF: fibroblast growth factor
Gli‐1: glioma 1
HRP: horseradish peroxidase
iFGF23: intact fibroblast growth factor 23
Npc1: niemann‐pick c1
PMF: primary myocardial fibroblast
Ptchd: patched‐related protein
SD: Sprague‐Dawley
Shh: Sonic Hedgehog
TGF: transforming growth factor

**Clinical Perspective**

**What Is New?**

- GANT‐61 had no significant effect on the degree of myocardial hypertrophy and hypertension in the Sprague‐Dawley rat 5/6 nephrectomy model.
- GANT‐61 improved cardiac function and reduced cardiac weight by inhibiting myocardial fibrosis.
- Elevated serum levels of intact fibroblast growth factor 23 in chronic kidney disease model rats promoted the excessive proliferation of cardiac fibroblasts and their transdifferentiation into myofibroblasts, leading to the synthesis and secretion of a large amount of extracellular matrix proteins, thereby promoting cardiac fibrosis.

**What Are the Clinical Implications?**

- As shown in the present study, ameliorating myocardial fibrosis in uremic cardiomyopathy improved cardiac function, providing some hope for clinically prolonging the life span of uremic patients.
- The Sonic Hedgehog signaling pathway and intact fibroblast growth factor 23 may be new therapeutic targets for delaying chronic kidney disease–induced cardiac fibrosis in the future.

Chronic kidney disease (CKD) is a highly prevalent disease, with a prevalence of ≈12% in the global population.^1^ Clinically, cardiovascular disease is one of the most important causes of death of patients with CKD on dialysis.^2^ CKD is a well‐known independent cardiovascular risk factor.^3^ Hypertension and cardiac hypertrophy are common in patients with CKD and are associated with increased mortality.^3^, ^4^ Cardiovascular diseases include heart disease. Numerous studies have shown that many heart diseases progress to the late stage of myocardial fibrosis.^5^ As a result, a large number of patients with CKD develop myocardial fibrosis.^6^ Overall, patients with heart disease and CKD are more likely to have more fibrotic tissue than patients with heart disease who are not diagnosed with CKD.^7^ Thus, myocardial fibrosis is an important cardiovascular complication in patients with CKD. Myocardial fibrosis is defined as an abnormal increase in fibrous connective tissue in cardiac tissue.^8^ The common features of fibrosis are the formation and aggregation of large amounts of connective tissue.^9^ Therefore, myocardial fibrosis is common in patients with fibrotic diseases. Transforming growth factor (TGF)‐β1 is an important profibrotic growth factor present in both cardiac and renal fibrosis. TGF‐β1 is a proven inducer of endothelial‐to‐mesenchymaltransition, and an interesting speculation is that kidney‐derived TGF‐β1 is involved in the myocardial fibrosis observed in the hearts of patients with CKD. Fibroblast growth factor (FGF) 2, a typical member of the FGF family, has long been presumed to stimulate the proliferation of fibroblasts and subsequently promote fibrosis.^10^ According to recent studies, hedgehog signaling is an important mediator of the development of renal fibrosis.^11^ However, activated hedgehog signaling in the heart has been shown to promote the formation of new blood vessels that maintain left ventricular function, thereby protecting myocardial cells.^12^ Nonetheless, activated hedgehog signaling in cardiac fibroblasts might promote myocardial fibrosis, reflecting the dual nature of hedgehog signaling. Transmitting a relatively stable signal, hedgehog plays an important role in maintaining tissue differentiation, regeneration, and repair. These effects of hedgehog signaling are outcomes of a series of hedgehog signaling‐induced cellular responses, such as proliferation, apoptosis, and differentiation.^13^ The classic hedgehog signaling pathway in mammals consists of 3 types of hedgehog ligands (namely, Sonic Hedgehog [Shh], Indian hedgehog, and Desert hedgehog). Two downstream transmembrane receptors have been identified: Smoothened and Patch.^14^ Recent studies have shown that Desert hedgehog is secreted only by gonad tissues in mammals, and Indian hedgehog is secreted in small amounts from some tissues, such as bone tissues. Shh is the most widely distributed hedgehog ligand, and it is secreted in large quantities by the heart tissue.^14^, ^15^ Therefore, in this study, Shh signaling was the main focus. When 1 of the 3 aforementioned secreted hedgehog ligands binds to a downstream Patch receptor, the hedgehog signaling pathway is activated.^16^ In humans, Patch is encoded by 7 homologous genes (namely, Patch 1, Patch 2, Ptchd1, Ptchd2, Ptchd3, Npc1, and Ptchd4).^16^ Among these receptors, Patch 1 is the main receptor in the hedgehog signaling pathway.^16^ Binding of Shh to Patch 1 releases Smoothened inhibition, leading to the accumulation and activation of Smoothened in cilia, which triggers downstream signaling.^17^, ^18^ Smoothened activation causes downstream glioma transcription factors to be activated, and activated glioma enters the nucleus and transmits signals by binding to the promoter of the target DNA, ultimately leading to specific gene expression.^14^, ^19^, ^20^, ^21^ Three proteins in the glioma family have been identified in vertebrates (namely, glioma 1 [Gli‐1], glioma 2, and glioma 3).^22^ The activity of the hedgehog signaling pathway is best reflected by the expression of Gli‐1.^23^ Therefore, Gli‐1 was selected as the indicator of hedgehog signaling pathway activation in this study. FGF23 is a phosphorylated hormone produced mainly by osteocytes and osteoblasts.^24^ An important role of the kidneys is to maintain normal serum calcium and phosphorus levels. However, in patients with CKD, normal serum phosphorus levels are not maintained; they are increased.^25^ High serum phosphorus levels in patients with CKD result in an increase in serum FGF23 levels.^26^ On the basis of accumulating evidence, an elevated serum FGF23 level is a marker of cardiovascular risk and may indicate potential mechanisms of cardiovascular disease in patients with CKD.^27^, ^28^, ^29^, ^30^ A causal relationship between elevated serum FGF23 levels in patients with CKD and the development of left ventricular hypertrophy was recently reported.^31^ Researchers have not clearly determined whether FGF23 plays a role in myocardial fibrosis caused by CKD, despite the findings of recent research. Currently, 2 methods are available for measuring FGF23 concentrations. The intact FGF23 (iFGF23) assay detects only biologically active full‐length FGF23.^32^ The C‐terminal FGF23 assay detects full‐length FGF23 and the degraded C‐terminal fragment of FGF23.^33^ We chose to observe iFGF23 in this study. Therefore, we hypothesized that an elevated serum level of iFGF23 plays a role in myocardial fibrosis caused by CKD and that hedgehog signaling promotes myocardial fibrosis in this process. In summary, we speculated that elevated serum levels of iFGF23 regulate CKD‐induced myocardial fibrosis by activating the hedgehog signaling pathway.

In this study, we sought to determine whether iFGF23 regulates CKD‐induced myocardial fibrosis by activating the hedgehog signaling pathway using a mature 5/6 nephrectomy (5/6NX) Sprague‐Dawley (SD) rat model of CKD and analyzed primary myocardial fibroblasts (PMFs) in vitro.

## METHODS

The data that support the findings of this study are available from the corresponding author on reasonable request.

### Animal Experiments

Male SD rats were randomly assigned to 3 groups: the sham operation group, the CKD+vehicle group, and the CKD+GANT‐61 (MedChemExpress, Monmouth Junction, NJ) group, with 6 rats in each group. After adaptive feeding for 1 week, the SD rats in the CKD+vehicle group and CKD+GANT‐61 group, which were 7 weeks old, were subjected to 5/6NX. During the procedure, 1 kidney was first completely removed. At 1 week after 1 kidney was completely removed, two‐thirds of the remaining kidney in each SD rat was removed, leaving only one‐third of a kidney in each rat. The sham group underwent the same operation, except that nephrectomy was not performed. One week later, these 9‐week‐old male SD rats underwent the next experiment.^34^ GANT‐61, a Gli‐1 inhibitor, dissolved in vehicle (5% dimethyl sulfoxide–PBS mixed solution) was injected intraperitoneally at 40 mg/kg into the CKD+GANT‐61 group of rats 3 times a week for 16 weeks. Rats in the sham operation group and CKD+vehicle group were administered a vehicle intraperitoneal injection 3 times a week for 16 weeks. All male SD rats were obtained from the Animal Experimental Center of Chongqing Medical University (Chongqing, China). Sixteen weeks after the injection, the SD rats were euthanized, the blood and hearts were collected for further analysis, and the mass of the hearts of these rats was also measured. Blood pressure measurements were performed at 0 (baseline), 8, and 16 weeks after surgery using a rat noninvasive sphygmomanometer (Softron, Tokyo, Japan). Before euthanasia, rats were placed in metabolic cages to obtain 24‐hour urine samples. The rats were then weighed. All animal experiments were conducted in accordance with the standards advanced by the Ethics Committee for Laboratory Animals of Chongqing Medical University, and the Ethics Committee for Laboratory Animals of Chongqing Medical University approved the experiments.

### Hematoxylin‐Eosin Staining of Myocardial Tissue

Myocardial tissue sections were deparaffinized, dehydrated, and stained with a hematoxylin solution for 5 minutes. Sections were then rinsed with tap water for 5 minutes, soaked in 1% hydrochloric acid in ethanol for 4 to 5 seconds, rinsed with tap water, and rinsed with distilled water for 5 minutes. The cytoplasm was stained by incubating sections with a 0.5% eosin solution for 2 minutes, and then the sections were gently washed with running tap water for 5 minutes. After staining, slides were dehydrated in an ethanol gradient for 3 minutes, clarified in xylene I, xylene II, and xylene III for 3 to 5 minutes, sealed with neutral balsam, and air dried. The scanned images of sections from each SD rat captured from 5 random fields of view at a magnification of ×400 were compiled using Pannoramic Scanner software. Cardiomyocyte diameters in the images were measured with ImageJ software.

### Sirius Red Staining and Quantification of Myocardial Fibrosis

The ventricular tissue was fixed with 4% paraformaldehyde and then embedded in paraffin. The samples embedded in wax blocks were placed in a paraffin microtome and cut into paraffin sections with a thickness of 3 to 4 μm for Sirius red staining. Sections from each rat were scanned using a biopsy scanner (Pannoramic DESK, Hungary), which produced full‐field digital images of the sections, to detect myocardial fibrosis. The scanned images of samples from each SD rat captured from 5 random fields of view at a magnification of ×200 were compiled using Pannoramic Scanner software. The collagen fibers in the images were quantified with ImageJ software.

### Immunofluorescence Staining of Myocardial Tissue

The prepared paraffin wax blocks were sliced into sections, as described previously. BSA (Servicebio, Wuhan, China) was added dropwise to the sections, and then, the sections were incubated for 30 minutes. The following primary antibodies were added to the sections: rabbit anti‐rat Smoothened antibody (1:200; Affinity), rabbit anti‐rat Gli‐1 antibody (1:100; Novus, CO), rabbit anti‐rat collagen‐1 antibody (1:200; Servicebio), and rabbit anti‐rat collagen‐3 antibody (1:200; Proteintech, Wuhan, China). The sections were placed flat inside a wet box and incubated overnight at 4 °C. The next day, the tissues were covered with secondary antibody corresponding to the primary antibody at a specific dilution and incubated at room temperature for 50 minutes. Horseradish peroxidase (HRP)–conjugated goat anti‐rabbit IgG was added as a secondary antibody to the samples (1:200; Servicebio).

Finally, the samples were sealed with a fluorescence quencher. The slices were placed under a scanner (Pannoramic DESK) to capture images, and the fluorescence intensity of each image was analyzed using ImageJ software.

### Immunohistochemical Staining of Myocardial Tissue

The paraffin sections were blocked with serum and incubated overnight with the following primary antibodies at 4 °C: rabbit anti‐rat Smoothened antibody (1:200; Affinity), rabbit anti‐rat Gli‐1 antibody (1:100; Novus), rabbit anti‐rat collagen‐1 antibody (1:1500; Servicebio), and rabbit anti‐rat collagen‐3 antibody (1:1000; Proteintech). The next day, the sections were incubated at room temperature with HRP‐conjugated goat anti‐rabbit IgG (1:200; Servicebio). Finally, the slices were placed under a scanner (Pannoramic DESK) to capture images. The positive area ratio of the images was determined with ImageJ software.

### Determination of Renal Function and Serum TGF‐β1, FGF2, iFGF23, NT‐proBNP, and Troponin I Levels in SD Rats

Blood urea nitrogen and serum creatinine: First, a working reagent was prepared according to the instructions provided with the kit (Cchuili, Changchun, China). Next, the stored serum samples were loaded into an automatic biochemical analyzer (Rayto Chemray 240, Shenzhen, China) and measured. Finally, the resulting data were exported into Excel software. Urinary albumin (mg/L) and creatinine (mmol/L) levels were determined with an automated clinical chemistry analyzer (Roche cobras integra 400 plus, Basel, Switzerland), and the urinary albumin/creatinine ratio was calculated. An ELISA kit (Abcam, Cambridge, UK) was used to determine the serum TGF‐β1 level in the SD rats. The test was performed in strict accordance with the manufacturer's instructions. An ELISA kit (R&D System, Minneapolis, MN) was used to determine the serum FGF2 level according to the manufacturer's instructions. The serum iFGF23 concentration in the SD rats was determined using an FGF23 ELISA kit (Cloud‐Clone Corp, Wuhan, China) for the detection of full‐length FGF23. The test was performed in strict accordance with the manufacturer's instructions. Serum NT‐proBNP (N‐terminal pro‐B‐type natriuretic peptide) levels were detected using an ELISA (R&D Systems). Serum troponin I levels were determined using an ELISA (Abcam).

### Isolation and Culture of Neonatal SD Rat Cardiac Fibroblasts

Neonatal SD rats from 1 to 3 days old were disinfected with alcohol under aseptic conditions and euthanized by cervical dislocation. After the heart tissues were removed, the heart tissues were cut into small pieces and fully digested with type 2 collagenase (Sigma Aldrich) and trypsin (Beyotime, Shanghai, China). The cells were then inoculated into a cell culture flask. Finally, cardiac fibroblasts were cultured in complete medium consisting of 10% fetal bovine serum (Gibco) and 90% F12 medium (Gibco) and placed in a 37 °C cell incubator containing carbon dioxide.

### Cell Stimulation and Plasmid Transfection

Myocardial fibroblasts were cultured in complete medium (F12 medium containing 10% fetal bovine serum). A plasmid (GeneChem, Shanghai, China) was transfected when cell confluence reached 80% to 90%. First, the cells were separated into 3 groups to verify the effectiveness of transfection of a negative control (NC) plasmid, a plasmid carrying the Shh gene (Shhp), and a plasmid carrying an RNA interference sequence targeting the Shh gene (Shh‐RNAi). After transfection, the cells were cultured in serum‐free medium (Opti‐MEM; Gibco). After 6 hours, the medium was changed to complete medium. After 42 hours, the cells were collected for further treatment. Then, the cells were separated into 5 groups. One group was transfected with a negative control plasmid (CON079), another group was transfected with an Shh gene‐carrying plasmid (54483–1), and another group was transfected with an Shh‐RNAi–carrying plasmid (76496–1). The 2 other groups were not transfected. All 5 groups were cultured in serum‐free medium. Six hours after transfection, the medium was changed to complete medium. After a total of 18 hours of cell culture, 10 ng/mL recombinant human FGF23 (PeproTech, NJ) was added to the NC plasmid‐expressing cells, Shhp‐expressing cells, Shh‐RNAi–transfected cells, and one group of untransfected cells; the other group of untransfected cells was treated with the same amount of complete medium as the transfected cells. Then, the cells were cultured for 48 hours in the presence of iFGF23 protein, and the medium of the control cells was replaced with the same amount of complete medium. After 48 hours of incubation, the cells were collected for further analysis.

### Real‐Time Quantitative Polymerase Chain Reaction Analysis of the Cells

The treated PMFs were collected. Total RNA was extracted with Invitrogen TRIzol reagent (Thermo Fisher, Harbin, China), according to the manufacturer's instructions. Next, the RNA was reverse transcribed into cDNAs with a reverse transcription kit (RR047A; Takara Bio, Japan). The following primer sequences were used for the real‐time quantitative polymerase chain reaction experiment: β‐actin forward, 5′‐ACGGTCAGGTCATCACTATCG‐3′; β‐actin reverse, 5′‐GGCATAGAGGTCTTTACGGATG‐3′; α‐smooth muscle actin (α‐SMA) forward, 5′‐GCATCCGACCTTGCTAACG‐3′; α‐SMA reverse, 5′‐CCAGAGTCCAGCACAATACCAG‐3′; collagen‐1 forward, 5′‐CAAGAAGTCCCTGCTCCTCCA‐3′; collagen‐1 reverse, 5′‐TGTGACTCGTGCAGCCATCC‐3′; collagen‐3 forward, 5′‐CAAGAGCGGAGAATACTGGGTT‐3′; collagen‐3 reverse, 5′‐TGGGACTGGCATTTATGCATG‐3′; Shh forward, 5′‐CTTTAGCCTACAAGCAGTTTATCC‐3′; Shh reverse, 5′‐TTGTGATCTTCCCTTCATATCG‐3′; Patch 1 forward, 5′‐TCTCACAACCCTCGGAACC‐3′; Patch 1 reverse, 5′‐CGACAGGGAAG‐3′; Gli‐1 forward, 5′‐AACTATGGCCCTGGCCACTGT‐3′; and Gli‐1 reverse, 5′‐CACCCTTGTTCTGGTTTTACTTGC‐3′. All primers were designed and synthesized by Takara Biotechnology Co, Ltd (Japan). Finally, an SYBR kit (RR820A; Takara Bio, Japan) was used for the polymerase chain reaction analysis of reverse‐transcribed cDNAs in 8‐strip tubes. After the samples were added, the polymerase chain reaction results were obtained with a CFX96 Touch Temperature Circulator (Bio‐Rad).

### Western Blot Analysis of Cellular Proteins

The treated PMFs were lysed with radioimmunoprecipitation assay buffer (Beyotime) containing the protease inhibitor phenylmethylsulfonyl fluoride (Beyotime). The protein concentration was measured according to the instructions of a bicinchoninic acid protein assay kit (Beyotime). Equal volumes of protein samples were loaded onto a 10% SDS‐PAGE gel (Beyotime), electrophoretically separated, and transferred to polyvinylidene difluoride membranes (Millipore, MA). After blocking with 5% skim milk at room temperature for 2 hours, the membranes were incubated overnight with the following primary antibodies at 4 °C: anti–β‐actin (1:3000; Servicebio), anti–α‐SMA (1:1000; Servicebio), anti–collagen‐1 (1:1000; Servicebio), anti–collagen‐3 (1:1000; Proteintech), anti‐Shh (1:1000; Proteintech), anti‐Smoothened (1:1000; Affinity), and anti–Gli‐1 (1:1000; Novus). Then, the membranes were incubated with the following secondary antibodies for 1 hour at room temperature: HRP‐conjugated anti‐mouse secondary antibody (1:5000; Servicebio) and HRP‐conjugated anti‐rabbit secondary antibody (1:5000; Servicebio). The protein bands were visualized using HRP‐substrate chemiluminescence technology (Servicebio). For quantification, the intensity of each target protein band was normalized to that of the β‐actin band.

### Cell Proliferation Assay

First, 5 groups of cells were grown in 6‐well plates. The NC plasmid and Shhp and Shh‐RNAi plasmids were transfected as described above. After 24 hours, the transfected cells were plated in a 96‐well plate at a density of 5000 cells per well in triplicate. In addition, intact FGF23 was added to the wells at a concentration of 10 ng/mL and incubated with the transfected cells for 48 hours. Then, Cell Counting Kit‐8 (Dojindo, Japan) reagent was added to cells in each well of the 96‐well plate and incubated for 4 hours. The absorbance of each well in the 96‐well culture plate was read at 450 nm with a microplate analyzer (Thermo, Finland). The absorbance value was used to reflect the proliferation of the cells in each group.

### Statistical Analysis

All data in this article are presented as the means±SEMs. GraphPad Prism 7 software was used to generate charts, and all statistical analyses were performed using SPSS 20.0 software. One‐way ANOVA was used to analyze data that met the preconditions of ANOVA, and the Kruskal‐Wallis test was used otherwise.

## RESULTS

### Renal Function and Related Indexes in SD Rats With 5/6NX

**Figure 1:**
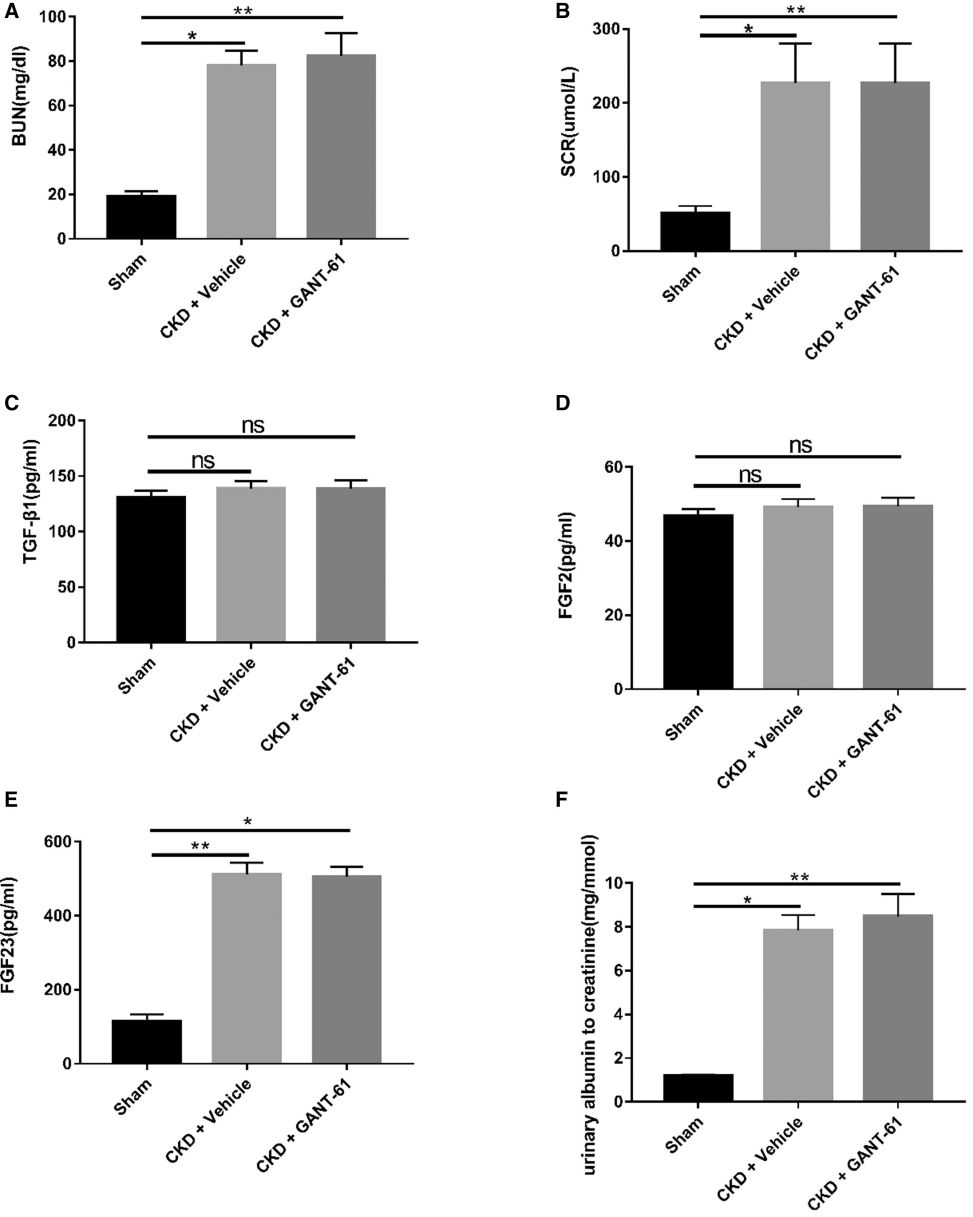
Renal function and serum‐related indexes changed in Sprague‐Dawley rats after 5/6 nephrectomy. Blood samples were collected 16 weeks after surgery. **A**, Blood urea nitrogen (BUN) levels (n=6 rats/group, *P<0.05 and **P<0.01). **B**, Serum creatinine (SCR) levels (n=6 rats/group, *P<0.05 and **P<0.01). **C** and **D**, Serum transforming growth factor (TGF)‐β1 and fibroblast growth factor (FGF) 2 levels (n=6 rats/group, not significant [ns]). **E**, FGF23 levels in the chronic kidney disease (CKD)+vehicle group and CKD+GANT‐61 group were higher than those in the sham operation group (n=6 rats/group, *P<0.05 and **P<0.01). **F**, Urinary albumin/creatinine ratio (n=6 rats/group, *P<0.05 and **P<0.01).

We determined whether the CKD model of SD rats was successfully established and whether the serum iFGF23, TGF‐β1, and FGF2 levels had changed by measuring the levels of the corresponding markers in serum. Blood urea nitrogen and serum creatinine levels in the SD rats of the 5/6NX group were significantly increased (Figure 1A and 1B). In addition, the urinary albumin/creatinine ratio in SD rats in the 5/6NX group was also significantly increased (Table and Figure 1F). Therefore, the CKD model was deemed to be successfully established. The serum FGF2 and TGF‐β1 contents in SD rats from the 5/6NX group did not change (Figure 1C and 1D). As described in the study by Sarlinova et al, elevated serum TGF‐β1 levels are not always observed in patients with CKD, indicating that the correlation between the 2 is uncertain.^35^ Therefore, according to the results of this experiment, kidney‐derived TGF‐β1 and FGF2 were not involved in myocardial fibrosis in the CKD model. Serum levels of FGF23 have been shown to be elevated in patients with CKD.^36^ In our study, the serum iFGF23 concentration in the SD rats of the 5/6NX group was increased (Figure 1E). According to the results of this study, the serum iFGF23 level was increased in the SD rat CKD model.

**Table .** Test Results From 5/6NX Rats

Parameters	Sham	CKD+vehicle	CKD+GANT‐61
Final body weight, g	563.68±9.38	555.8±9.89	532.45±6.63
Heart weight, mg	1523.17±292.83	3013.50±142.50	2376.67±130.63
Relative heart weight, mg/g	2.71±0.55	5.42±0.32	4.46±0.26
UACR, mg/mmol	1.19±0.44	7.84±0.70	8.48±1.02
SBP, mm Hg			
Baseline	114.09±5.47^ns^	111.92±8.77	113.75±10.10^ns^
At wk 8	116.33±6.57*	144.18±9.59	143.86±12.04^ns^
At wk 16	118.88±4.01*	146.33±5.50	144.67±8.29^ns^
DBP, mm Hg			
Baseline	75.55±4.97^ns^	74.96±4.21	76.11±5.27^ns^
At wk 8	78.61±8.49*	114.11±6.96	112.84±7.47^ns^
At wk 16	79.00±7.10*	116.00±9.42	114.83±10.34^ns^

Normally distributed data are presented as the mean±SD. n=6 rats/group. 5/6NX, 5/6 nephrectomy; CKD indicates chronic kidney disease; DBP, diastolic blood pressure; ns, not significant; SBP, systolic blood pressure; and UACR, urinary albumin/creatinine ratio.

* *P*<0.001 compared with the CKD+vehicle groups. Multiple comparisons were performed using 1‐way ANOVA followed by the Fisher least significance difference (LSD) test.

### Effects of 5/6NX and GANT‐61 on Myocardial Fibrosis Levels in SD Rats

**Figure 2:**
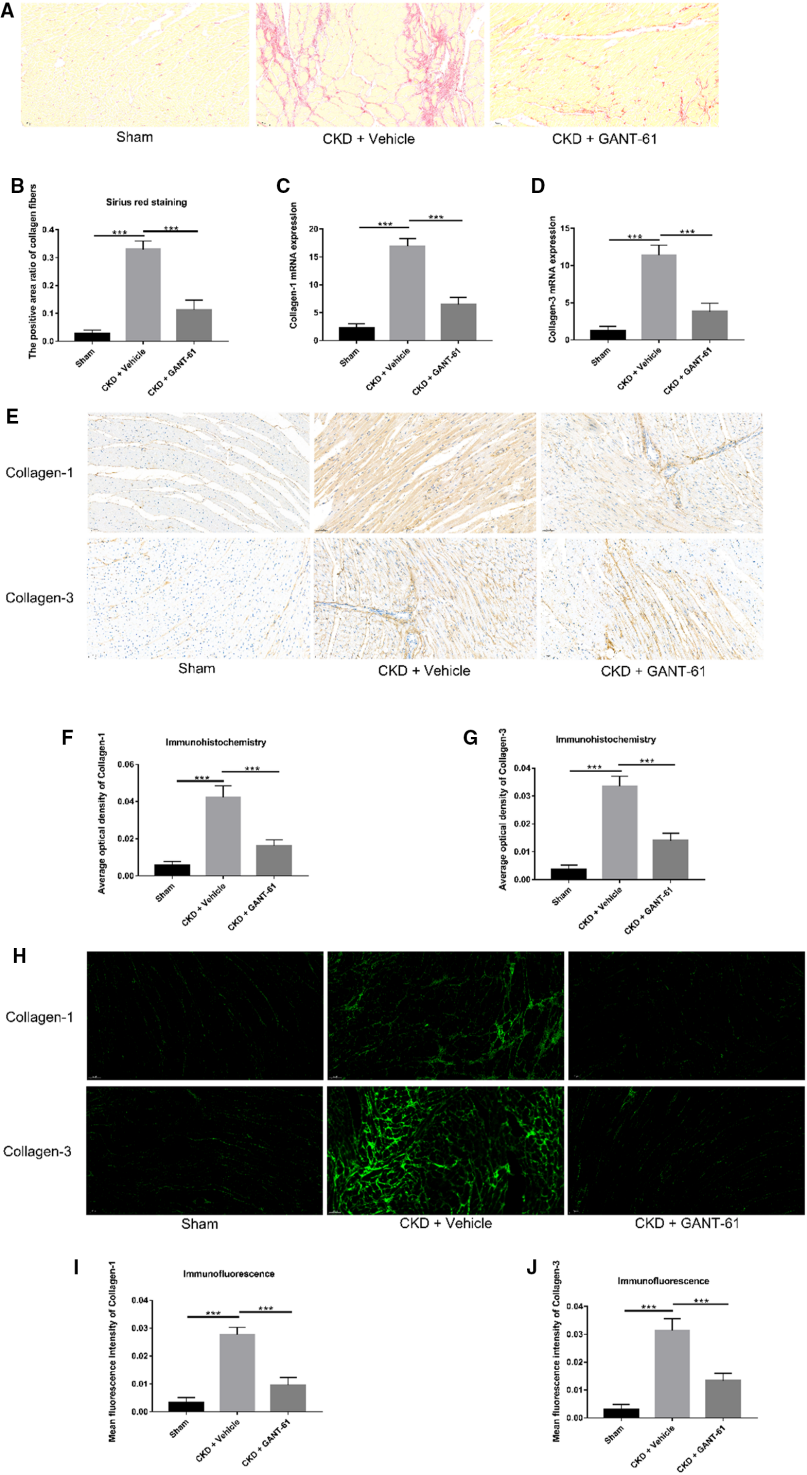
Effects of 5/6 nephrectomy and GANT‐61 on myocardial fibrosis levels in Sprague‐Dawley (SD) rats. Hearts were collected 16 weeks after surgery. **A** and **B**, Sirius red staining in the myocardial tissue of SD rats (n=6 rats/group, ***P<0.001, bar=50 μm). **C** and **D**, Real‐time quantitative polymerase chain reaction analysis of collagen‐1 and collagen‐3 expression in SD rat myocardial tissue (n=6 rats/group, ***P<0.001). **E** through **G**, Immunohistochemistry in the myocardium of SD rats. The average optical density (OD) values of collagen‐1 and collagen‐3 in the myocardial tissue sections from the SD rats in the chronic kidney disease (CKD)+vehicle group were significantly higher than those in the SD rats from the sham group. Compared with the CKD+vehicle group, the average OD values of collagen‐1 and collagen‐3 in myocardial tissue sections from SD rats in the CKD+GANT‐61 group were decreased (n=6 rats/group, ***P<0.001, bar=50 μm). **H** through **J**, Immunofluorescence staining in the cardiac tissue of SD rats. The average fluorescence intensity of collagen‐1 and collagen‐3 in the myocardial tissue in the rats of the CKD+vehicle group was higher than that in the sham group. The average fluorescence intensity of collagen‐1 and collagen‐3 in myocardial tissue sections of SD rats in the CKD+GANT‐61 group was decreased compared with that in the CKD+vehicle group (n=6 rats/group, ***P<0.001, bar=50 μm).

First, Sirius red staining was used to assess the level of myocardial fibrosis in the SD rats. In Figure 2A, myocardial fiber deposition was significantly increased in SD rats from the CKD+vehicle group. Fibrotic tissue deposition in the myocardium of 5/6NX SD rats was ameliorated in the presence of the Gli‐1 inhibitor GANT‐61. The positive area ratio of the collagen fibers was used to calculate the difference in myocardial fibrosis of the myocardial tissue in different rat groups, and as shown in Figure 2B, CKD caused myocardial fibrosis. The degree of myocardial fibrosis in SD rats with CKD was attenuated in the presence of GANT‐61, a Gli‐1 inhibitor of the Hedgehog signaling pathway. In Figure 2C and 2D, collagen‐1 and collagen‐3 mRNA levels were increased in CKD rats in the CKD+vehicle group. Compared with the CKD rats in the CKD+vehicle group, the CKD rats in the CKD+GANT‐61 group exhibited decreased mRNA levels of collagen‐1 and collagen‐3 in the presence of the inhibitor GANT‐61. Next, we performed immunohistochemistry to evaluate the levels of collagen‐1 and collagen‐3 in the myocardium of the SD rats. In Figure 2E through 2G, the brown area in the myocardium of SD rats in the CKD+vehicle group was significantly larger than that in the sham‐operated group. The brown area in the myocardium of SD rats in the CKD+GANT‐61 group was reduced compared with that in the CKD+vehicle group. The average optical density of the immunohistochemically stained sections was used to calculate the difference in the levels of collagen‐1 and collagen‐3 in the myocardial tissue from different rat groups. The levels of collagen‐1 and collagen‐3 in the myocardium of SD rats in the CKD+vehicle group were higher than those in the sham group. After administering the inhibitor GANT‐61, the levels of collagen‐1 and collagen‐3 in the myocardium of the SD rats in the CKD+GANT‐61 group were decreased compared with those in the CKD+vehicle group. Finally, immunofluorescence staining was performed to analyze the levels of collagen‐1 and collagen‐3 in the heart tissue of SD rats. In Figure 2H through 2J, the average fluorescence intensity was measured to calculate the difference in the levels of collagen‐1 and collagen‐3 in different rat groups. In the immunofluorescently stained sections of SD rat myocardial tissue, collagen‐1 and collagen‐3 levels were increased in SD rats from the CKD+vehicle group. However, the levels of collagen‐1 and collagen‐3 were decreased in SD rats from the CKD+GANT‐61 group after GANT‐61 was administered. In summary, the results of the 4 measurements all suggest that the degree of myocardial fibrosis in SD rats from the CKD+vehicle group was higher than that in the sham operation group. However, the degree of myocardial fibrosis in SD rats in the CKD+GANT‐61 group was lower than that in the CKD+vehicle group. Therefore, we propose that CKD causes myocardial fibrosis and that the degree of myocardial fibrosis caused by CKD is reduced after the use of the Gli‐1 inhibitor GANT‐61, which targets the hedgehog signaling pathway.

### Effects of 5/6NX and GANT‐61 on the Expression Level of Components of the Hedgehog Pathway in SD Rats

**Figure 3:**
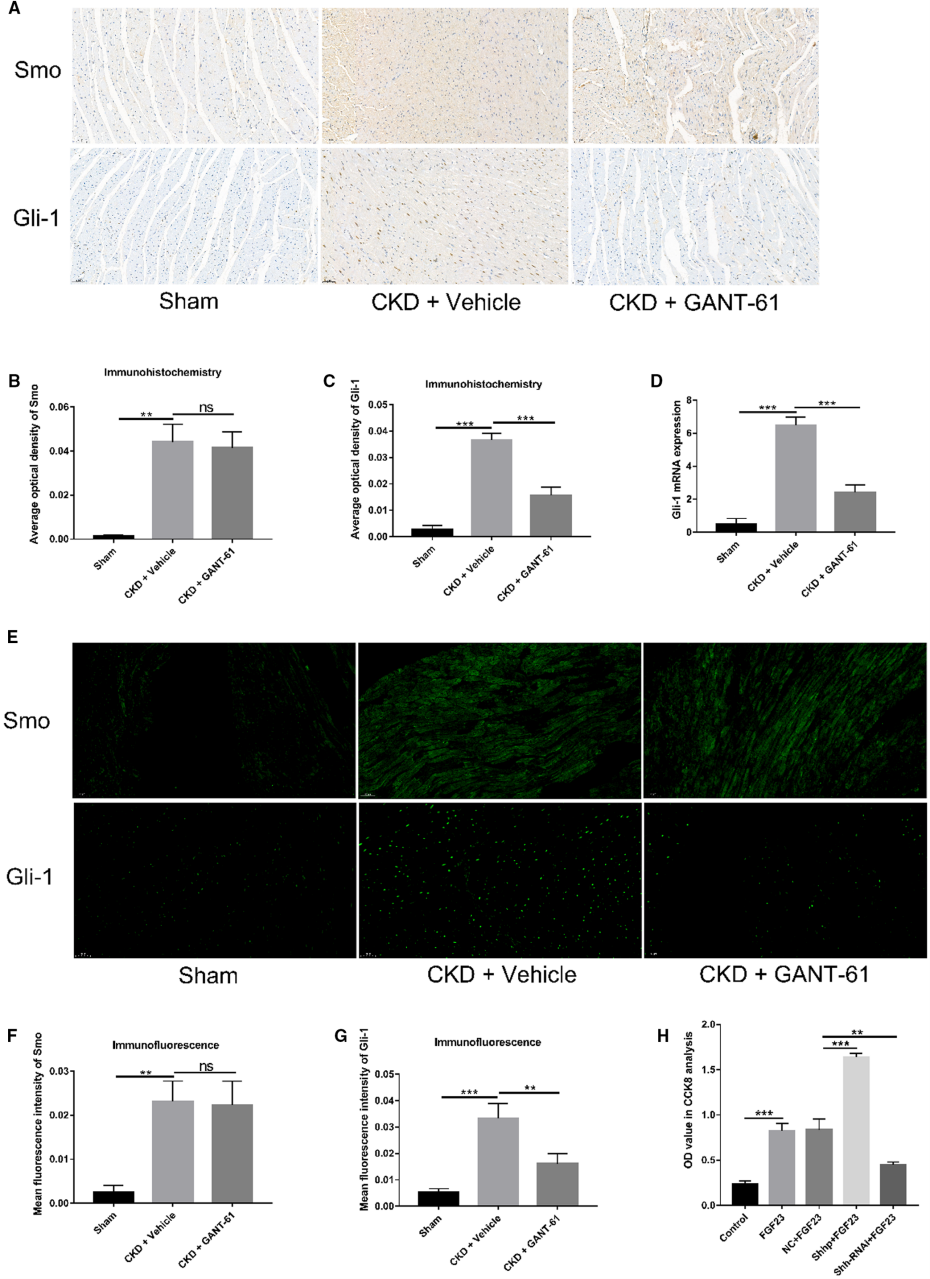
Effects of 5/6 nephrectomy and GANT‐61 on the expression level of components of the hedgehog (Hh) pathway in Sprague‐Dawley (SD) rats and Cell Counting Kit‐8 (CCK‐8) assay using cardiac fibroblasts. **A** through **C**, Immunohistochemistry in the myocardium of the SD rats (n=6 rats/group, not significant [ns], **P<0.01, and ***P<0.001; bar=50 μm). **D**, Real‐time quantitative polymerase chain reaction analysis of glioma 1 (Gli‐1) expression in SD rat myocardial tissues (n=6 rats/group, ***P<0.001). **E** through **G**, Immunofluorescence staining in the myocardium of the SD rats (n=6 rats/group, ns, **P<0.01, and ***P<0.001; bar=50 μm). **H**, Fibroblast growth factor (FGF) 23 promoted the proliferation of primary myocardial fibroblasts in SD rats by activating the Sonic Hh (Shh) signaling pathway. The optical density (OD) value represents the number of cells (n=3 rats/group, **P<0.01 and ***P<0.001). CKD indicates chronic kidney disease; NC, negative control; Shhp, plasmid carrying the Shh gene; Shh‐RNAi, plasmid carrying an RNA interference sequence targeting the Shh gene; and Smo, Smoothened.

The hedgehog pathway is critical for tissue remodeling and is involved in fibrotic diseases in many parts of the body.^37^ Recent evidence shows that hedgehog signaling is closely related to the occurrence and development of cardiovascular diseases.^38^ Therefore, we investigated whether the hedgehog signal was increased in the fibrotic myocardium of SD rats in the CKD+vehicle group compared with SD rats in the sham‐operated group. At the same time, we determined whether the expression of Gli‐1 in the hedgehog signaling pathway was inhibited after treatment with the inhibitor GANT‐61. Among the factors measured, Gli‐1 and Smoothened are the 2 key factors downstream in the hedgehog signaling pathway. We performed immunohistochemistry and immunofluorescence staining to determine whether the expression of the indicators Gli‐1 and Smoothened increased in the fibrotic myocardium of SD rats from the CKD+vehicle group. At the same time, we detected whether the increase in Gli‐1 expression was inhibited in SD rats from the CKD+GANT‐61 group. In Figure 3A through 3C, the difference in the expression levels of Smoothened and Gli‐1 among the 3 groups was calculated using the average optical density of the immunohistochemically stained myocardial slices. The expression levels of Gli‐1 and Smoothened in the fibrotic myocardium of rats in the CKD+vehicle group were higher than those of rats in the sham operation group. Compared with the CKD+vehicle group, the Gli‐1 expression level in the fibrotic myocardium of the CKD+GANT‐61 group was decreased. In Figure 3D, the Gli‐1 mRNA level was higher in the myocardial tissue of SD rats in the CKD+vehicle group than that in SD rats in the sham‐operated group. However, the mRNA level of Gli‐1 in the myocardial tissue of SD rats in the CKD+GANT‐61 group was lower than that in the CKD+vehicle group. In Figure 3E through 3G, the average fluorescence intensity of the immunofluorescently stained sections was used to calculate the differences in the expression levels of Gli‐1 and Smoothened among the 3 groups. Compared with SD rats in the sham operation group, the expression levels of Gli‐1 and Smoothened in the fibrotic myocardium of SD rats in the CKD+vehicle group were increased. Lower Gli‐1 expression was detected in the myocardial tissue of the CKD+GANT‐61 group than in the CKD+vehicle group. Therefore, we speculate that the hedgehog signaling pathway is activated in the fibrotic myocardium of the SD rats in the CKD+vehicle group. The use of the inhibitor GANT‐61 reduces the expression of Gli‐1, thereby inhibiting the hedgehog signaling pathway.

### Hematoxylin‐Eosin Staining, Blood Pressure, and Other Related Indicators in SD Rats

**Figure 4:**
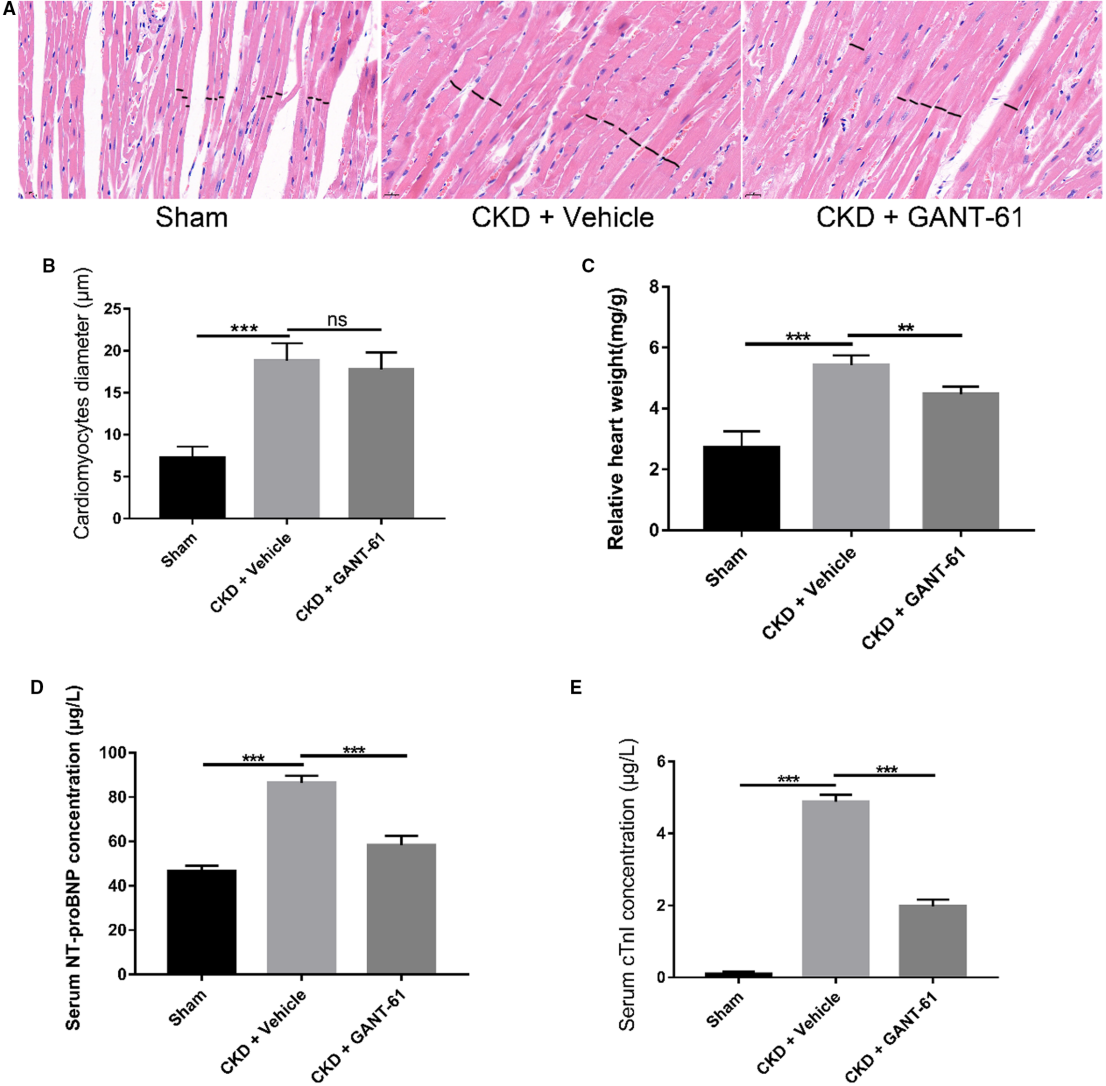
Hematoxylin‐eosin (HE) staining, blood pressure, relative heart weight, and cardiac function biomarkers in Sprague‐Dawley (SD) rats. **A** and **B**, HE staining and cardiomyocyte diameter (n=6 rats/group, not significant [ns] and ***P<0.001; bar=20 μm). **C**, Relative heart weight (n=6 rats/group, **P<0.01 and ***P<0.001). **D** and **E**, Serum NT‐proBNP (N‐terminal pro‐B‐type natriuretic peptide) and troponin I (TnI) levels in SD rats (n=6 rats/group, ***P<0.001). CKD indicates chronic kidney disease.

The final body weight, heart weight, and relative heart weight of SD rats are shown in the Table. Relative heart weight was increased in rats from the CKD+vehicle group compared with rats from the sham group. The relative heart weight of rats in the CKD+GANT‐61 group was reduced compared with that of rats in the CKD+vehicle group (Figure 4C). Next, we performed hematoxylin‐eosin staining to observe the morphological changes in the rat myocardial tissue. Compared with the sham group of rats, the myocardial fibers of the CKD+vehicle group of rats were thickened and exhibited a disordered arrangement, some edges were blurred, and obvious myocardial hypertrophy was detected. Similar findings were obtained for the myocardial tissue of rats in the CKD+GANT‐61 group (Figure 4A). We quantified the degree of cardiac hypertrophy by calculating the cardiomyocyte diameter. Compared with that of the sham group, the cardiomyocyte diameter was significantly increased in the CKD+GANT‐61 group and the CKD+vehicle group. However, a significant difference in cardiomyocyte diameter was not observed between the CKD+GANT‐61 group and the CKD+vehicle group (Figure 4B). Therefore, our results suggest that GANT‐61 may not affect the diameter of cardiomyocytes, possibly reducing cardiac weight by shortening the length of cardiomyocytes. It is also possible that, given the limitations of hematoxylin‐eosin staining, we did not observe changes in cardiomyocyte diameter. By measuring the systolic blood pressure (mm Hg) and diastolic blood pressure (mm Hg) of the rats, we did not observe a difference in blood pressure among groups at 0 weeks after surgery. Blood pressure levels in the CKD+GANT‐61 group and the CKD+vehicle group increased at 8 weeks after surgery and were almost maintained at the 8‐week levels at 16 weeks after surgery. A significant difference in blood pressure was not observed between the CKD+GANT‐61 group and the CKD+vehicle group at either 8 or 16 weeks after surgery. The blood pressure of rats in the sham‐operated group was maintained at a stable level from week 0 to week 16 after the operation (Table). On the basis of our results, we cannot yet conclude that GANT‐61 affects blood pressure. Finally, we detected the levels of NT‐proBNP and troponin I, 2 commonly used^39^ cardiac biomarkers (Figure 4D and 4E). On the basis of these findings, we speculate that GANT‐61 inhibits fibrosis and subsequently improves cardiac function. Of course, GANT‐61 may affect the indicators of cardiac function by acting on the kidneys and other factors, so herein we can only speculate that GANT‐61 may directly affect cardiac function.

### Transfection of Primary SD Rat Cardiac Fibroblasts With Shhp and Shh‐RNAi Plasmids Upregulated and Downregulated the Expression of Shh, Respectively

**Figure 5:**
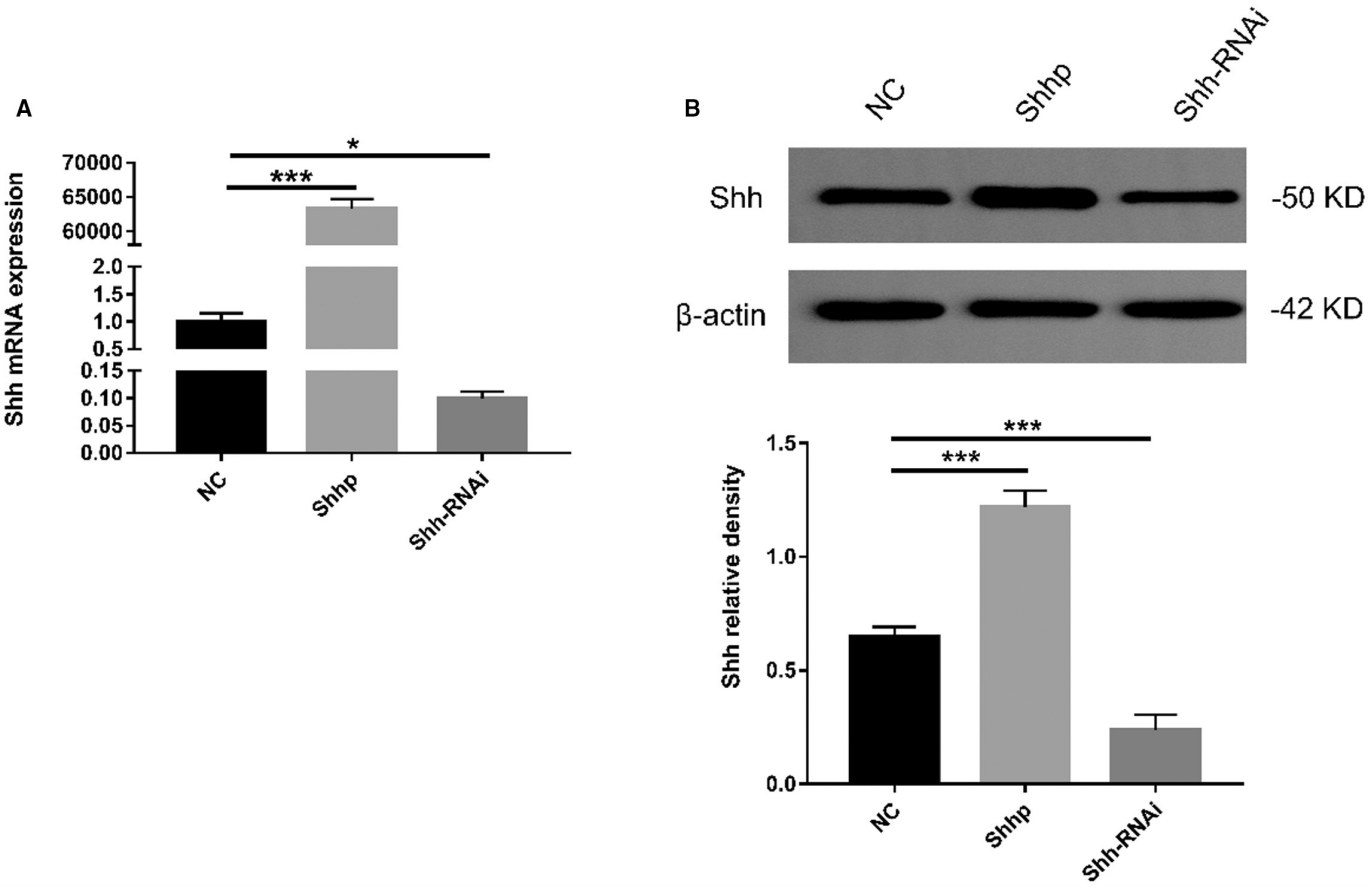
Transfection of Sprague‐Dawley rat primary myocardial fibroblasts with the plasmid carrying the Sonic Hedgehog (Shh) gene (Shhp) and plasmid carrying an RNA interference sequence targeting the Shh gene (Shh‐RNAi) upregulated and downregulated the expression of Shh, respectively. **A**, Real‐time quantitative polymerase chain reaction analysis of the cells (n=3 samples/group, *P<0.05 and ***P<0.001). **B**, Western blot analysis of the 3 groups (n=3 samples/group, ***P<0.001). NC indicates negative control.

As mentioned above, 3 hedgehog ligands have been identified in mammals. Among these ligands, Shh has been the most extensively researched and exhibits the highest expression in the heart.^40^, ^41^ In addition, studies have shown that an injection of Shhp into mice upregulates the Shh signaling pathway.^12^ Therefore, Shh is the main upstream gene in the Shh signaling pathway, and we studied its effects on pathways and the fibrotic phenotype by upregulating and downregulating its expression level. As shown in Figure 5A, compared with that in the NC plasmid group, Shh mRNA expression in the Shhp group increased significantly. Compared with the NC plasmid group, the expression of the Shh mRNA in the Shh‐RNAi plasmid group was significantly reduced. Thus, the Shhp and Shh‐RNAi plasmids exert clear regulatory effects on the mRNA level. As shown in Figure 5B, compared with the NC plasmid group, Shh protein levels in the Shhp group increased significantly. Compared with the NC plasmid group, Shh protein expression in the Shh‐RNAi plasmid group was significantly reduced. These findings show that the Shhp and Shh‐RNAi plasmids exert clear regulatory effects on the translational level. In summary, the Shhp and Shh‐RNAi plasmids exert clear regulatory effects on transcription and translation.

### iFGF23 Induces Increased Levels of Myocardial Fibrosis Markers in Vitro by Activating the Shh Signaling Pathway

**Figure 6:**
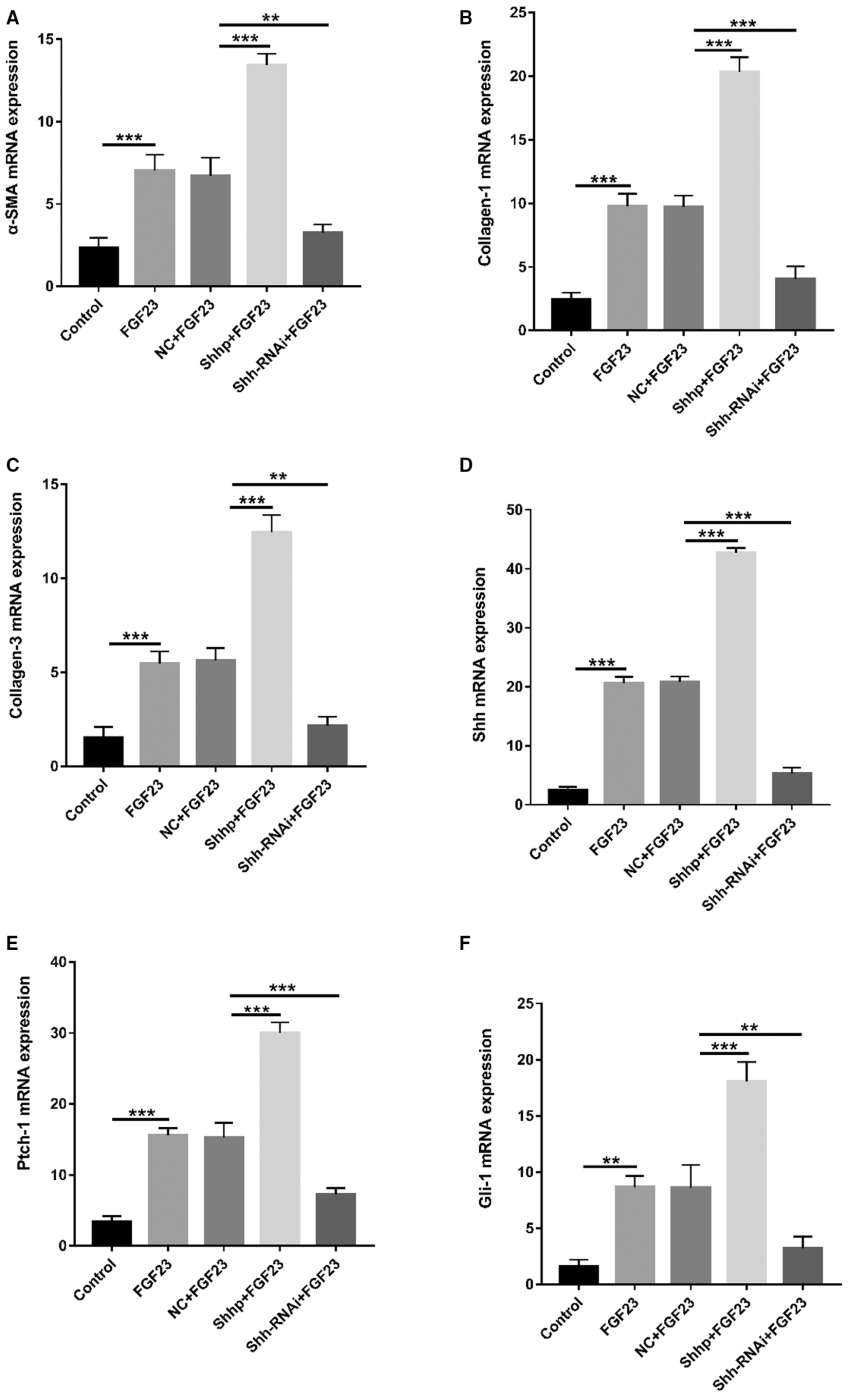
Fibroblast growth factor (FGF) 23 increases the levels of myocardial fibrosis markers in vitro by activating the Sonic Hedgehog (Shh) signaling pathway. **A** through **F,** The mRNA levels of α‐smooth muscle actin (α‐SMA), collagen‐1, collagen‐3, Shh, Patch 1 (Ptch‐1), and glioma 1 (Gli‐1) in the FGF23 group were significantly increased compared with those in the control group. No significant differences were observed between the negative control (NC)+FGF23 group and the FGF23 group. The mRNA levels of α‐SMA, collagen‐1, collagen‐3, Shh, Ptch‐1, and Gli‐1 in the plasmid carrying the Shh gene (Shhp)+FGF23 group were significantly higher than those in the NC+FGF23 group. The mRNA levels of α‐SMA, collagen‐1, collagen‐3, Shh, Ptch‐1, and Gli‐1 in the plasmid carrying an RNA interference sequence targeting the Shh gene (Shh‐RNAi)+FGF23 group were significantly decreased compared with those in the NC+FGF23 group (n=3 samples/group, **P<0.01 and ***P<0.001). Cells were primary myocardial fibroblasts.

**Figure 7:**
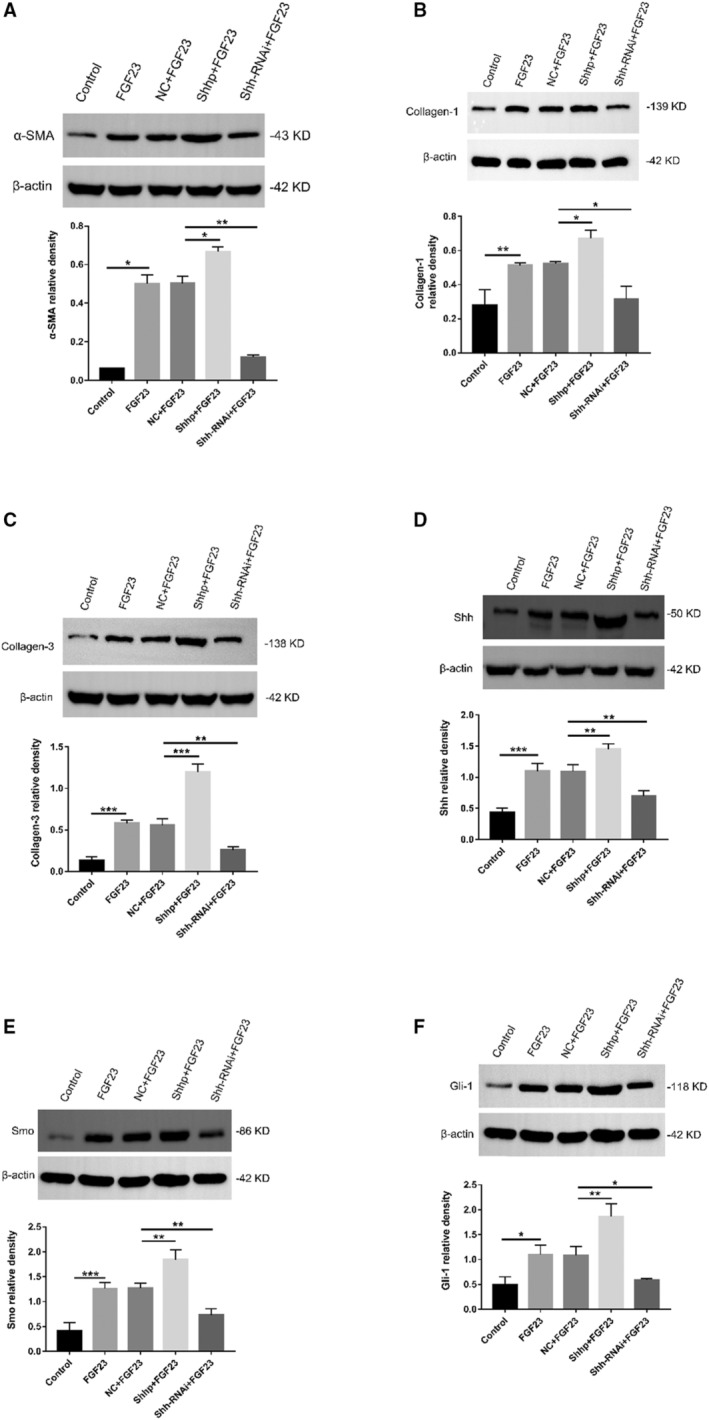
Fibroblast growth factor (FGF) 23 increases the levels of myocardial fibrosis markers in vitro by activating the Sonic Hedgehog (Shh) signaling pathway. **A** through **F**, The protein levels of α‐smooth muscle actin (α‐SMA), collagen‐1, collagen‐3, Shh, Smoothened (Smo), and glioma 1 (Gli‐1) in the FGF23 group were significantly increased compared with those in the control group. No significant differences were observed between the negative control (NC)+FGF23 group and the FGF23 group. Levels of the α‐SMA, collagen‐1, collagen‐3, Shh, Smo, and Gli‐1 proteins in the plasmid carrying the Shh gene (Shhp)+FGF23 group were significantly higher than those in the NC+FGF23 group. α‐SMA, collagen‐1, collagen‐3, Shh, Smo, and Gli‐1 protein levels in the plasmid carrying an RNA interference sequence targeting the Shh gene (Shh‐RNAi)+FGF23 group were significantly decreased compared with those in the NC+FGF23 group (n=3 samples/group, *P<0.05, **P<0.01, and ***P<0.001). Cells were primary myocardial fibroblasts.

Elevated FGF23 levels are associated with many cardiovascular conditions, including cardiac hypertrophy, atherosclerosis, vascular calcification, and endothelial dysfunction.^31^, ^42^, ^43^, ^44^, ^45^ In addition, elevated FGF23 levels are related to cardiovascular events in patients with CKD. Therefore, we used the iFGF23 protein to stimulate SD rat PMFs and determine whether it increased the extent of intracellular fibrosis and/or whether it activated the Shh signaling pathway. Then, we transfected SD rat PMFs with the Shh plasmid and Shh‐RNAi plasmid to upregulate and downregulate Shh expression levels. We further evaluated whether Shh signaling pathway components were upregulated or downregulated to determine whether the Shh signaling pathway promoted an increase in the degree of fibrosis in cardiac fibroblasts. As shown in Figure 6A through 6F, iFGF23 regulated the increase in fibrosis in SD rat PMFs at the transcriptional level in vitro, promoted cellular fibrosis at the transcriptional level, and activated the Shh signaling pathway. In addition, the plasmid itself did not interfere with the experimental results obtained at the transcriptional level. Shhp upregulated the expression of components of the Shh signaling pathway at the transcriptional level, and activation of the Shh signaling pathway promoted fibrosis in SD rat PMFs at the transcriptional level. Finally, the Shh‐RNAi plasmid downregulated Shh signaling pathway components, and downregulation of Shh signaling pathway components inhibited fibrosis in SD rat PMFs at the transcriptional level. As shown in Figure 7A through 7F, iFGF23 promoted fibrosis in SD rat PMFs at the translational level in vitro and activated the Shh pathway to promote cellular fibrosis. In addition, the plasmid itself did not interfere with the experimental results obtained at the translational level. Subsequently, the Shh plasmid increased the expression of proteins in the Shh signaling pathway at the translational level. Next, the increased levels of proteins in the Shh signaling pathway promoted fibrosis in SD rat PMFs at the translational level. Finally, the Shh‐RNAi plasmid increased the levels of proteins in the Shh signaling pathway, and decreased levels of proteins in the Shh signaling pathway inhibited the fibrosis of PMFs in SD rats at the translational level. In summary, iFGF23 promoted fibrosis in PMFs from SD rats by activating the Shh signaling pathway.

### iFGF23 Promotes the Proliferation of PMFs in SD Rats by Activating the Shh Signaling Pathway

Combined with the previously obtained results from the α‐SMA assay, we performed a Cell Counting Kit‐8 assay to determine whether iFGF23 promoted the proliferation of SD rat PMFs by activating the Shh signaling pathway, thereby promoting fibroblast transdifferentiation into myofibroblasts. As shown in Figure 3H, iFGF23 promoted the proliferation of PMFs from SD rats. Combined with previously obtained experimental results, iFGF23 stimulation increased the expression of the myofibroblast‐specific marker α‐SMA in cells. We speculate that iFGF23 promotes the proliferation of SD rat PMFs and ultimately promotes the transdifferentiation of SD rat PMFs into SD rat myofibroblasts. In iFGF23‐stimulated cells, the plasmid itself had no effect on cell proliferation. Next, we transfected Shhp into iFGF23‐stimulated cells, and Shhp activated the Shh signaling pathway. Therefore, we suggest that the activation of the Shh signaling pathway promotes the proliferation of SD rat PMFs. Considering the results presented herein and our previous experimental results, we conclude that activation of the Shh signaling pathway promotes the transdifferentiation of SD rat PMFs by inducing their proliferation. Finally, on stimulation with iFGF23, the Shh‐RNAi plasmid downregulated Shh signaling pathway components. As mentioned above and based on our previous findings, we suggest that the downregulation of Shh signaling pathway components inhibited myocardial fibroblast proliferation, thereby inhibiting their transdifferentiation. In summary, iFGF23 promotes the proliferation of SD rat PMFs by activating the Shh signaling pathway, thereby further promoting their transdifferentiation into myofibroblasts.

## DISCUSSION

In this study, we first assessed the degree of myocardial fibrosis in a classic model of CKD. Our results confirmed that CKD may cause myocardial fibrosis. Then, we found that components of the Shh signaling pathway related to the degree of myocardial fibrosis were upregulated in the hearts of CKD model rats. Using GANT‐61, an inhibitor of the Shh signaling pathway component Gli‐1, we observed an attenuation of CKD‐induced myocardial fibrosis. We measured the serum iFGF23 concentration in the CKD model SD rats, as well as the serum concentrations of TGF‐β1 and FGF2, which are associated with fibrosis. The serum iFGF23 concentration was elevated, whereas the serum concentrations of TGF‐β1 and FGF2 did not change significantly. Thus, renal‐derived iFGF23 may be related to myocardial fibrosis caused by CKD, and renal‐derived TGF‐β1 and FGF2 were not found to be involved in myocardial fibrosis caused by CKD. Finally, we examined the degree of cardiac hypertrophy, cardiac weight, blood pressure, and cardiac function biomarkers in SD rats. On the basis of these results, GANT‐61 had no significant effect on the diameter of hypertrophic myocardium and the degree of hypertension in the 5/6 SD rat nephrectomy model. We speculate that GANT‐61 may improve cardiac function by inhibiting myocardial fibrosis, and may reduce cardiac weight by reducing the length of hypertrophic cardiomyocytes. In vitro, iFGF23 activated the Shh signaling pathway in cardiac fibroblasts during cardiac fibrosis. Then, considering the stimulation of cardiac fibroblasts by iFGF23, we upregulated and downregulated the expression of Shh signaling pathway components and found that the Shh signaling pathway was positively correlated with the degree of cardiac fibrosis. In addition, the Shh signaling pathway promoted the excessive proliferation of cardiac fibroblasts and their transdifferentiation into myofibroblasts. According to the results obtained after the inhibition of the Shh signaling pathway in vivo and the upregulation and downregulation of the Shh pathway in vitro, we postulate that the Shh signaling pathway is involved in the process of myocardial fibrosis caused by CKD, and interventions targeting Shh signaling are a feasible treatment option. At the same time, combining the results of the in vivo and in vitro experiments, we concluded that elevated iFGF23 in the serum of CKD model rats promotes the excessive proliferation of cardiac fibroblasts and their transdifferentiation into myofibroblasts, leading to the synthesis and secretion of a large amount of extracellular matrix (ECM) proteins, thereby promoting cardiac fibrosis. In summary, we concluded that elevated serum iFGF23 levels in the CKD model promote the proliferation and transdifferentiation of cardiac fibroblasts by activating the Shh signaling pathway, thereby promoting cardiac fibrosis. This conclusion is consistent with our initial hypothesis.

Fibrosis is an important cause of organ dysfunction in many diseases, including interstitial lung disease, liver cirrhosis, diabetic nephropathy, and heart failure.^46^ Myocardial fibrosis is a serious complication of CKD.^47^ The main characteristic of cardiac fibrosis is activated cardiac fibroblasts that proliferate and differentiate into myofibroblasts, which produce and secrete a large amount of ECM material, creating a large deposition of ECM.^48^, ^49^, ^50^ The excessive accumulation of ECM proteins causes the expansion of the heart interstitium, which is evident in most cardiac conditions.^51^, ^52^ Therefore, myocardial fibrosis leads to serious consequences, such as increased heart stiffness and impaired diastolic filling.^53^, ^54^ The ECM is mainly composed of fibrous filaments composed of collagen, among which collagen‐1 and collagen‐3 are the most abundant.^55^, ^56^, ^57^, ^58^ When fibroblasts are activated and transdifferentiated into myofibroblasts, myofibroblasts, in particular, express α‐SMA, as manifested by increased expression levels of α‐SMA.^59^, ^60^ In addition, 2 ECM proteins, collagen‐1 and collagen‐3, are secreted and are thus markers of cardiac fibrosis.^58^, ^61^ Therefore, in our study, collagen‐1 and collagen‐3 levels were used as markers of myocardial fibrosis. α‐SMA levels were also used as a marker to detect the extent of cardiac fibroblast fibrosis and further clarify whether treatment promoted the transdifferentiation of cardiac fibroblasts. CKD is an important cause of cardiovascular disease.^62^ A previous study showed that FGF23 binds to FGF receptor 4 in cardiomyocytes to promote cardiomyocyte hypertrophy.^63^ In addition, Hao et al found that FGF receptor 4 is expressed in cardiac fibroblasts and that its expression increased during myocardial ischemia.^64^ Notably, our results indicate that iFGF23 induced myocardial fibrosis by stimulating myocardial fibroblasts, suggesting that iFGF23 may bind to FGF receptor 4 on myocardial fibroblasts and eventually lead to myocardial fibrosis. This hypothesis is consistent with the results reported by Böckmann et al.^65^ According to Chu et al, the C‐terminal fragment of FGF23 is also biologically active.^66^ However, our study only suggests that full‐length FGF23 is involved in the process of myocardial fibrosis caused by uremia. We have not determined whether the C‐terminal fragment of FGF23 is involved in the process of myocardial fibrosis caused by uremia. It may be involved in the myocardial fibrosis caused by uremia, but the mechanism may be completely different from that of full‐length FGF23. Zeng et al found that sodium‐glucose co‐transporter 2 blockers significantly inhibit cardiac fibrosis in 5/6NX model rats and reduce hypertension levels.^67^ However, the Gli‐1 inhibitor we studied unfortunately did not attenuate hypertension while inhibiting myocardial fibrosis in the CKD model. Gli‐1 inhibitors are also clinically promising treatments for inhibiting CKD‐induced myocardial fibrosis.

Many studies have shown that the Shh signaling pathway is highly active during fibrotic disease pathogenesis in many parts of the body, suggesting a potential relationship between fibrosis in many parts of the body and abnormal Shh signaling.^68^, ^69^, ^70^ According to recent studies, the Shh signaling pathway is activated after renal injury and is the most important mediator of the progression of renal injury to renal fibrosis.^11^, ^68^ The hedgehog pathway is also involved in a variety of liver injury conditions, including liver fibrosis, inflammation‐related injuries, and liver cancer.^71^, ^72^ In addition, some studies have found that the Shh signaling pathway is one of the most important signaling pathways involved in the development of pulmonary fibrosis.^73^ Therefore, we believe that the Shh signaling pathway is involved in the occurrence and development of myocardial fibrosis and promotes the progression of myocardial fibrosis. Interestingly, Kusano et al showed that the Shh signaling pathway promotes angiogenesis, nourishing the myocardium and inhibiting cardiac fibrosis induced by acute myocardial infarction.^12^ Notably, Kusano et al studied cardiac fibrosis following acute myocardial infarction. In contrast, we examined fibrosis of the heart resulting from CKD. Therefore, the differences between our study and the work of Kusano et al are notable. Cardiac fibrosis caused by CKD is continuous.^7^ Specifically, the key process in cardiac fibrosis results from scar formation after acute myocardial infarction, whereas pathological fibrosis is a continuous process that involves all areas of the heart and is often referred to as remodeling.^54^, ^74^ Therefore, we drew a different conclusion than Kusano et al. In addition, Kusano et al did not directly study the relationship between the Shh signaling pathway and cardiac fibroblasts. Therefore, we cannot exclude the possibility that the Shh signaling pathway promotes the transdifferentiation of myocardial fibroblasts after acute myocardial infarction, promoting cardiac scarring. The role of the Shh signaling pathway in myocardial fibroblast transdifferentiation is possibly obscured by its role in heart muscle nourishment in the early stages of disease, and the Shh signaling pathway may play a more dominant role in some chronic diseases over time; thus, its effects are identified only on the late course of a disease. This possibility is worthy of further exploration.

One of the main shortcomings of our study is that we did not measure whether more substances in the serum of SD rats in the CKD model were abnormal. In addition to the 2 typical indicators that cause fibrosis listed above, many substances are present in the serum of CKD rats. These substances, such as various uremic toxins, may be involved in myocardial fibrosis caused by CKD. Because of the limited scope of our research, we aimed to clarify the role of iFGF23 because clarifying this part of the mechanism may guide the direction of clinical treatment to a certain extent. Many abnormal substances are present in the serum of patients with CKD. More research is needed in the future to reveal other potentially active substances. Another minor shortcoming is that not all components of the Shh signaling pathway were measured in the CKD model rats. However, the detection of 2 key genes, Smoothened and Gli‐1, downstream in the Shh signaling pathway may explain the findings. In vivo experiments showed that only the regulation of Gli‐1 modulated the Shh pathway. Similarly, our in vitro experiments regulating the upstream components of the Shh pathway also achieved the purpose of regulating the Shh pathway. These findings support our conclusion. The mechanism by which CKD leads to myocardial fibrosis is complex, and many aspects of CKD have not been extensively studied. Therefore, this research direction is interesting, and more research is needed to understand the mechanisms involved.

In conclusion, we confirmed that elevated iFGF23 serum levels are associated with the development of myocardial fibrosis in a CKD model. Our results also show that the Shh signaling pathway is activated in the process of myocardial fibrosis induced by CKD. In addition, iFGF23 participates in regulating the CKD process, leading to cardiac fibrosis by mediating the activation of the Shh signaling pathway. These findings identify the Shh signaling pathway and iFGF23 as possible new therapeutic targets for delaying CKD‐induced cardiac fibrosis in the future.

## Sources of Funding

This research was supported by the National Science and Technology Support “Twelfth Five‐Year” Project and the Ministry of Education's Special Research Fund for Doctoral Programs.

## Disclosures

None.
